# The ratio of intratumoral CD15 ^+^ neutrophils to CD8 ^+^ lymphocytes predicts recurrence in patients with gastric cancer after curative resection

**DOI:** 10.1002/cnr2.2099

**Published:** 2024-06-04

**Authors:** Junichiro Watanabe, Takashi Kimura, Zenichiro Saze, Naoya Sato, Yasuhide Kofunato, Teruhide Ishigame, Ryo Okada, Akira Kenjo, Koji Kono, Shigeru Marubashi

**Affiliations:** ^1^ Department of Hepato‐Biliary‐Pancreatic and Transplant Surgery Fukushima Medical University Fukushima City Japan; ^2^ Department of Gastrointestinal Tract Surgery Fukushima Medical University Fukushima City Japan

**Keywords:** CD15/CD8, gastric cancer, neutrophil‐to‐lymphocyte ratio, overall survival, relapse‐free survival

## Abstract

**Background:**

An elevated neutrophil‐to‐lymphocyte ratio (NLR) in peripheral blood is an independent prognostic indicator of various cancers.

**Aims:**

In this study, we aimed to investigate the prognostic relevance of the intratumoral immune cell balance in gastric cancer.

**Methods and Results:**

The study included 82 patients who underwent curative resection for gastric cancer. The intratumoral cluster of differentiation (CD) 15‐ and CD8‐positive cells were evaluated using immunohistochemical staining. Additionally, clinicopathological factors and prognoses were analyzed. Patients with high intratumoral CD15/CD8 ratios had significantly lower overall survival (OS) and relapse‐free survival (RFS) compared to those with low CD15/CD8 ratios (*p* = .0026 and *p* < .0001, respectively). Additionally, a high CD15/CD8 ratio was associated with lymph node metastasis (*p* = .019). Patients with high NLR had a significantly lower RFS than those with low NLR (*p* = .0050). Multivariate analysis revealed that the intratumoral CD15/CD8 ratio, NLR, and venous invasion were independent prognostic indicators of RFS (CD15/CD8 ratio: *p* < .001, hazard ratio (HR) = 14.7, 95% confidence interval (CI) = 3.8–56.8; NLR: *p* = .010, HR = 5.4, 95% CI = 1.5–19.6; venous invasion: *p* = .005, HR = 7.4, 95% CI = 1.8–29.7).

**Conclusion:**

In summary, we found that the intratumoral CD15/CD8 ratio is an independent prognostic factor following gastric cancer resection and its increase is associated with lymph node metastasis and microscopic lymph vessel invasion. Immunological evaluation with additional aspects of innate immunity may be useful in predicting cancer prognosis.

## 1 INTRODUCTION

Inflammation and the immunological environment within tumor tissues have been closely linked to tumor progression and prognosis. Current research is delving into the detailed mechanisms behind this association.^1^ Analysis of gene expression in a large cohort of cancer tissues, using samples from The Cancer Genome Atlas (TCGA), has revealed that the expression of immune‐related genes within the tumor environment ranks second in importance to cell cycle‐related genes across a broad spectrum of cancers.^2^ Moreover, prior studies have predicted the proportions of tumor‐infiltrating leukocytes based on increased gene expression in tumor tissues. These findings indicate that high expression of genes specific to certain lymphocyte subtypes, such as CD8‐positive lymphocytes, associated with a favorable prognosis.^2^, ^3^ In contrast, high expression of genes specific to myeloid leukocytes, like neutrophils, tends to predict a poor prognosis.^2^, ^4^, ^5^ This evidence suggests that the balance between key leukocytes namely CD8‐positive lymphocytes and neutrophils in tumor tissues could serve as a predictive marker for patient outcomes.

Treatment strategies for gastric cancer, from early‐stage to advanced cancers, have evolved through the development of detailed algorithms based on the results of numerous clinical studies. This has led to a constant improvement in treatment outcomes.^6^, ^7^, ^8^ By stratifying the risk of recurrence among patients who have undergone curative resection and offering more effective treatment to those at higher risk and less invasive treatment or follow‐up to those at lower risk, it is hoped to establish a treatment strategy that improves prognosis while also contributing to patient quality of life (QOL) and healthcare economics. To achieve this goal, it is important to examine the risk of recurrence in more detail.

Comprehensive immunogenomic analysis of TCGA samples suggested that gastric cancer has a high frequency of subtypes with high leukocyte infiltration, such as lymphocytes and macrophages, and that the immunologic landscape of the cancer tissue may influence prognosis.^3^ This study aims to investigate the prognostic value of the ratio of CD15‐positive neutrophils to CD8‐positive lymphocytes in tumor tissues from patients with resected gastric cancer through immunohistochemistry. Additionally, we analyzed clinicopathological associations to determine their potential impact on prognosis.

## 2 METHODS

### 2.1 Patient samples

This study included consecutive 82 patients who underwent curative surgery for gastric cancer at the Fukushima Medical University, Japan, from January 2009 to December 2011. Informed consent was obtained from all patients to collect and analyze specimens for this study. The study design was a retrospective cohort study, and ethics committee approval was obtained in 2014, after surgery. Inclusion criteria were all patients who underwent surgery with the intention of curative surgery for gastric cancer during the same period, and patients who were deemed ineligible for curative surgery at the time of surgery were excluded. Pathological findings were evaluated based on the 8th Edition of the TNM Classification of Gastric Cancer.

### 2.2 Immunohistochemistry

Following the standard protocol, immunohistochemical staining for CD15 and CD8 was performed on 4‐μm formalin‐fixed paraffin‐embedded tissue sections. After deparaffinization, the antigen was microwave‐activated for 15 min, and endogenous peroxidase activity was inhibited by incubation with 0.3% aqueous hydrogen peroxide for 20 min. The tissue samples were subsequently treated with 5% powdered skim milk to block nonspecific reactions with antibodies. Slides were incubated for 20 min at 4°C with the following primary antibodies: CD15 (1:50 dilution, mouse monoclonal, clone MMA, BD Biosciences, San Jose, CA, USA) and CD8 (1:50 dilution, mouse monoclonal, clone C8/144B, DAKO, Santa Clara, CA, USA). Slides were then incubated with biotinylated anti‐mouse secondary antibodies (rabbit polyclonal, 1:400 dilution; DAKO). Antigen visualization was performed using the VECTASTAIN Elite ABC Standard Kit (1:200 dilution; VECTOR Laboratories, San Diego, CA, USA). Immunostained slides were observed under an optical microscope (IX73, OLYMPUS, Japan) at 400× and images of this field of view were captured. CD15‐positive neutrophils and CD8‐positive lymphocytes in the field of view were counted visually, and the average value was used as the number of each positive cell. When identifying CD15‐positive cells, morphological characteristics were considered to exclude CD15‐positive eosinophils. The CD15/CD8 ratio was calculated by dividing the number of CD15‐positive cells by the number of CD8‐positive cells. Automated analysis using image analysis software was not used to identify positive cells. Images of the slides taken were displayed in ImageJ (https://imagej.net/ij/index.html) and counted manually one by one.

### 2.3 NLR of peripheral blood

Peripheral blood collected within 4 weeks prior to the surgery was analyzed. The total white blood cell counts and percentages of neutrophils and lymphocytes were retrieved from the electronic records of the hospital laboratory. NLR was defined as the absolute neutrophil count divided by the absolute lymphocyte count.

### 2.4 Statistical analysis

Statistical analyses were performed using IBM SPSS Statistics version 23 (IBM, Armonk, NY, USA) and GraphPad Prism, version 7.03 (GraphPad Software, San Diego, CA, USA). OS was defined as the period between the date of surgery and death, while RFS was defined as the period between the date of surgery and recurrence. The cutoff values for low and high NLR and CD15/CD8 ratio were determined using receiver operating characteristic (ROC) curve analysis. The survival curve was prepared using the Kaplan–Meier method, and statistical differences were analyzed using the log‐rank test. The association between the immune balance in tumor tissues and the clinicopathological factors was evaluated using the chi‐square or Fisher's exact test. The normality of the distribution of the CD15/CD8ratio and NLR variables was tested by the Kolmogorov–Smirnov test. The correlations were further analyzed by Spearman's rank correlation coefficient. Univariate and multivariate analyses were performed using the Cox proportional hazard model. The statistical significance for all analyses was set at *p* < .05.

## 3 RESULTS

### 3.1 Patient characteristics

The patient demographics and clinical characteristics are presented in Table 1. The mean age of the patients was 67.7 years. Although patients were predominantly male, neither age nor sex affected prognosis. None of the patients were treated with preoperative adjuvant chemotherapy. The median follow‐up time after surgery was 61 months (range: 4–94 months, interquartile range: 24). In addition to deaths, there were also cases in which follow‐up was lost during the course of the study and patients dropped out of follow‐up. In total, 21 patients died at the end of the follow‐up period. Among them, 14 patients died from cancer‐unrelated diseases, and 11 patients experienced tumor relapse. The median time to relapse was 12 months (range: 6–28 months, interquartile range: 13). Twenty‐four patients received postoperative adjuvant chemotherapy (Table 1).

**TABLE 1:** Patient demographic and clinical characteristics.

Factors	Number or period
All cases	82
Median follow‐up time	61 months (4–94, IQR; 34)
Tumor relapse	11 (12.6%)
Median time to relapse	12 months (6–28, IQR: 13)
Adjuvant chemotherapy	24 (27.5%)
Age	67.7 ± 11.2
Gender	
Male	63 (76.8%)
Female	19 (23.1%)
Depth of invasion	
T1a	26 (31.7%)
T1b	29 (35.4%)
T2	6 (7.3%)
T3	7 (8.5%)
T4a	14 (17.1%)
LN metastasis	
N0	56 (68.3%)
N1	8 (9.8%)
N2	6 (7.3%)
N3	12 (14.6%)
Staging	
I	54 (65.9%)
II	12 (14.6%)
III	16 (19.5%)
Histological grade	
Differentiated	39 (47.6%)
Undifferentiated	43 (52.4%)
Lymphatic invasion	
ly0	32 (39.0%)
ly1	23 (28.0%)
ly2	8 (9.8%)
ly3	19 (23.2%)
Venous invasion	
v0	41 (50.0%)
v1	15 (18.3%)
v2	10 (12.2%)
v3	16 (19.5%)
CEA (ng/mL)	
<5.0	67 (89.3%)
≧5.0	8 (10.7%)
CA19‐9 (U/mL)	
<37.0	68 (91.9%)
≧37.0	6 (8.1%)
ALB (g/dL)	
<3.8	32 (39.0%)
≧3.8	50 (61.0%)

Abbreviations: ALB, albumin; CA19‐9, carbohydrate antigen 19‐9; CEA, carcinoembryonic antigen; IQR, interquartile range; LN, lymph node.

### 3.2 Gastric cancer tissues with high intratumoral CD15‐positive cells have poor prognosis

**FIGURE 1:**
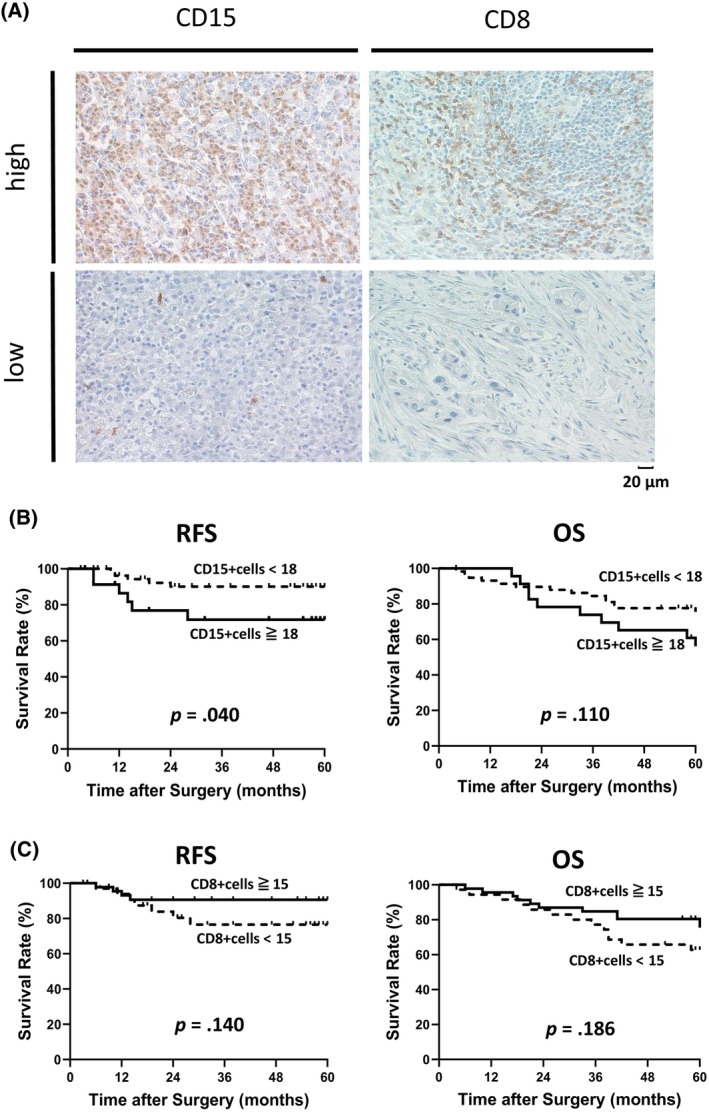
Relationship between the number of CD15‐positive and CD8‐positive cells and prognosis of gastric cancer. (A) Representative immunohistochemical tissue section of intratumoral infiltrating CD15‐positive and CD8‐positive cells. The image was observed with an optical microscope at 400×. (B) Kaplan–Meier curves showing RFS and OS for patients with gastric cancer based on CD15‐positive cells (*n* = 82). (C) Kaplan–Meier curves showing RFS and OS for patients with gastric cancer based on CD8‐positive cells (*n* = 82). CD, cluster of differentiation; OS, overall survival; RFS, relapse‐free survival.

Figure 1A shows representative cases with high and low CD15‐positive and CD8‐positive cells. As an example, in Figure 1A, there were 477 positive cells for CD15 high, 163 for CD8 high, 6 for CD15 low and 3 for CD8 low. The median number of CD15‐positive and CD8‐positive cells per field of view was 11 (range: 0–209) and 16 (range: 1–88), respectively. The median intratumoral CD15/CD8 ratio was 0.8 (range: 0–18.2). According to ROC curve analysis, the cutoff values for CD15‐ and CD8‐positive cells were 18 (Se, Sensitivity: 0.545; Sp, Specificity: 0.239; AUC, Area under the curve: 0.680) and 15 (Se: 0.364; Sp: 0.549; AUC: 0.412), respectively, based on the 5‐year RFS. Patients were divided into two groups: those with high CD15‐ and CD8‐positive cells and those with low CD15‐ and CD8‐positive cells (Figure 1B,C). Patients with high numbers of intratumoral CD15‐positive cells in gastric cancer tissues had poorer RFS than those with low numbers (RFS: *p* = .040, OS: *p* = .110). However, the number of intratumoral CD8‐positive cells did not affect prognosis (RFS: *p* = .140, OS: *p* = .186).

### 3.3 High intratumoral CD15/CD8 ratio worsens prognosis

**FIGURE 2:**
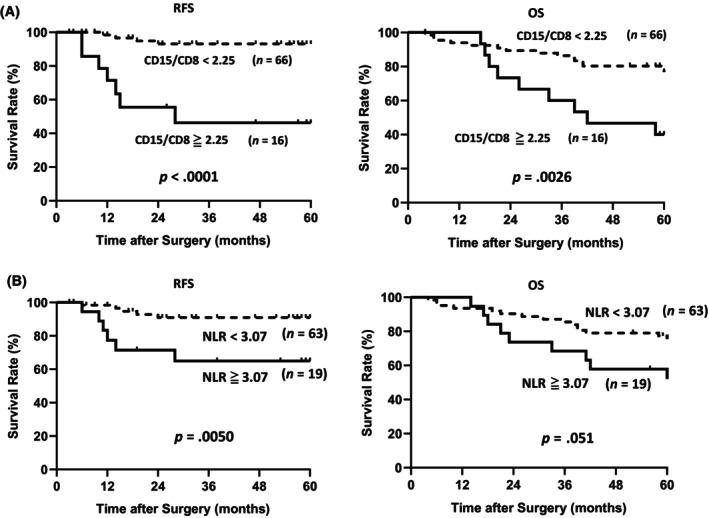
Relationship between NLR, RFS, OS, and CD15/CD8 ratio in gastric cancer patients. (A) Kaplan–Meier curve depicting RFS and OS for gastric patients divided by CD15/CD8 ratio (*n* = 82). (B) Kaplan–Meier curves showing RFS and OS for gastric patients divided by NLR (*n* = 82). CD, cluster of differentiation; NLR, neutrophil–lymphocyte ratio; OS, overall survival; RFS, relapse‐free survival.

ROC curve analysis revealed that the cutoff value for the CD15/CD8 ratio was 2.25 based on the 5‐year RFS, as depicted in Figure 2A. Patients were divided into two groups: patients with high CD15/CD8 ratios, and patients with low CD15/CD8 ratios. Patients with high intratumoral CD15/CD8 ratios had poorer RFS and OS than those with low ratios. The five‐year RFS in the low and high CD15/CD8 groups were 93.9% and 56.3%, respectively (*p* < .0001). The hazard ratio (HR) for RFS was 10.8 (HR: 3.2–37.2, *p* < .0001). The five‐year OS in the low and high CD15/CD8 groups were 77.3% and 43.8% (*p* = .0026), respectively. The HR for OS was 3.3 (HR: 1.4–7.6, *p* = .005).

### 3.4 High intratumoral CD15/CD8 ratio is associated with lymph node metastasis and lymphatic invasion in gastric cancer tissues

The demographic characteristics of each patient group are presented in Table 2 based on the investigated intratumoral CD15/CD8 ratio and NLR. Patients with a higher intratumoral CD15/CD8 ratio had a significantly higher incidence of lymph node metastasis (*p* = .019) and lymphatic invasion (*p* = .005). In contrast, the incidence of the depth of invasion (*p* = .024), lymph node metastasis (*p* < .001), staging (*p* = .013), and lymphatic invasion (*p* < .001) was considerably elevated in patients with higher NLRs.

**TABLE 2:** Intratumoral CD15/CD8 ratio and NLR in association to clinicopathologic factors.

Clinicopathological factors	CD15/CD8 ratio		*p*‐value	NLR		*p*‐value
	Low (<2.25)	High (≧2.25)		Low (<3.07)	High (≧3.07)	
All cases 82	66	16		63	19	
Age (median: 69, range: 31–89) <69	31	6	.495	31	6	.176
(IQR: 14) ≧69	35	10		32	13	
Gender						
Male	49	14	.259	47	16	.539
Female	17	2		16	3	
Depth of invasion						
T1a	23	3	.214	24	2	.024*
T1b–T4	43	13		39	17	
LN metastasis						
N0	49	7	.019*	51	5	<.001*
N1–N3	17	9		12	14	
Staging						
I	45	9	.367	46	8	.013*
II–III	21	7		17	11	
Histological grade						
Differentiated	31	8	.828	32	7	.286
Undifferentiated	35	8		31	12	
Lymphatic invasion						
ly0, ly1	49	6	.005*	50	5	<.001*
ly2, ly3	17	10		13	14	
Venous invasion						
v0, v1	47	9	.249	45	11	.266
v2, v3	19	7		18	8	
CEA (ng/mL)						
<5.0	55	12	1.000	54	13	.356
≧5.0	7	1		5	3	
CA19‐9 (U/mL)						
<37.0	55	13	.583	55	13	.111
≧37.0	6	0		3	3	
ALB (g/dL)						
<3.8	23	9	.115	21	11	.054
≧3.8	43	7		42	8	

*Note*: *p*‐values were estimated using chi‐squared test or Fisher's exact test *p < .05.

Abbreviations: ALB, albumin; CA19‐9, carbohydrate antigen 19‐9; CD, cluster of differentiation; CEA, carcinoembryonic antigen; LN, lymph node; IQR, interquartile range.

### 3.5 NLR and CD15/CD8 ratios are independent of each other

**FIGURE 3:**
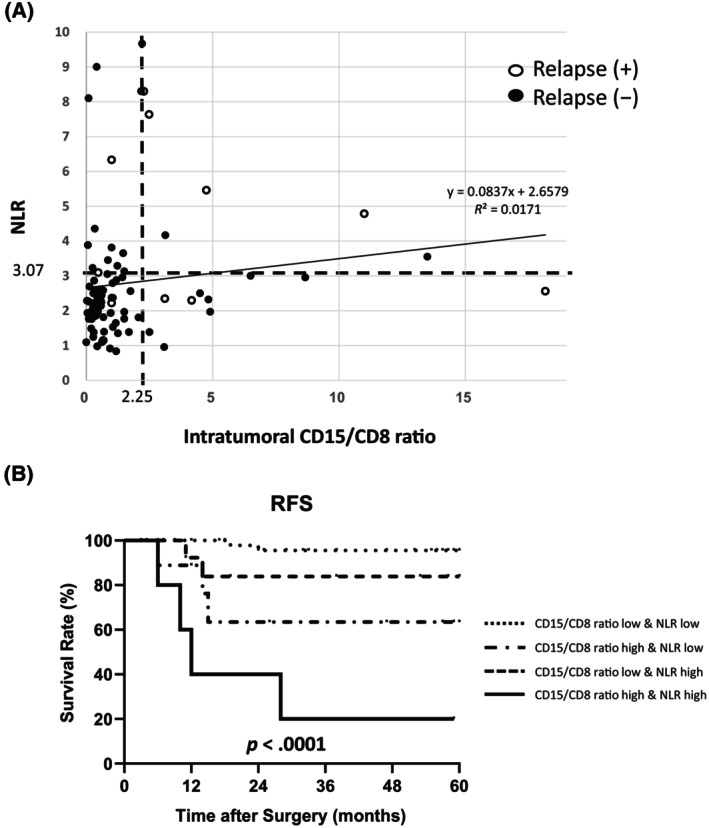
Comparison of prognosis by CD15/CD8 ratio and NLR combination. (A) Scatter plot of NLR and CD15/CD8 ratio of each patient. Cases with relapse were plotted as white circles, and those without relapse were plotted as black circles. (B) Comparison of prognosis by CD15/CD8 ratio and NLR combined with high and/or low. CD, cluster of differentiation; NLR, neutrophil–lymphocyte ratio; RFS, relapse‐free survival.

NLR was determined using preoperative peripheral blood data. The median value for the NLR was 2.29 (range: 0.83–9.67). According to the ROC curve, based on the 5‐year RFS, the optimal NLR cutoff value was 3.07. Patients with a high NLR had worse RFS (Figure 2B; *p* = .0050). The Kolmogorov–Smirnov test confirmed that both the CD15/8 ratio and NLR were not normally distributed with *p* < .0001. Therefore, the correlation analysis of the CD15/CD8 ratio and NLR was conducted using Spearman's rank correlation coefficient, a nonparametric test. The result showed that there was a correlation with a significance probability of *p* = .046, but the correlation coefficient was *ρ* = .221, which was a very weak correlation (Figure 3A).

Relapse occurred in 3 of the 29 patients with a high NLR or CD15/CD8 ratio, at a relapse rate of 31.0%. As a breakdown of this, relapse occurred in 3 of the 10 patients with a low NLR and high CD15/CD8 ratio, 2 of the 13 patients with a high NLR and low CD15/CD8 ratio, and 4 of the 6 patients with a high NLR and high CD15/CD8 ratio. In contrast, recurrence occurred in 2 of 53 patients with low NLR and CD15/CD8 ratio, at a relapse rate of 3.8%. Analysis of the difference in relapse rates among these four categories revealed a significantly higher relapse rate in the group with high NLR and high CD15/CD8 ratio (Figure 3B; *p* < .0001).

### 3.6 A high intratumoral CD15/CD8 ratio, NLR, and venous invasion are an independent prognostic factor for gastric cancer

RFS‐related factors were evaluated using univariate and multivariate analyses. These analyses also included albumin (ALB), and a component of prognostic scores, such as the Prognostic Nutritional Index and Glasgow Prognostic Score. Univariate analysis revealed a significant difference in intratumoral CD15/CD8 ratio (*p* < .001), NLR (*p* = .011), and venous invasion (*p* = .025). Multivariate analysis, which included the intratumoral CD15/CD8 ratio, NLR, and venous invasion, revealed that the intratumoral CD15/CD8 ratio (*p* < .001), NLR (*p* = .010), and venous invasion (*p* = .005) were independently associated with RFS (Table 3).

**TABLE 3:** Univariate and multivariate Cox proportional hazards regression analysis of clinicopathological factors for relapse‐free survival.

Clinicopathological factors	Univariate analysis		Multivariate analysis	
	Hazard ratio	*p*	Hazard ratio	*p*
Depth of invasion (T1a or T1b–T4)	37.6 (0.2, >1000)	.179		
Lymph node metastasis (N0 or N1–N3)	284.3 (0.5, >1000)	.077		
Histological grade (differentiated or undifferentiated)	2.4 (0.6, 8.9)	.204		
Lymphatic invasion (ly0, ly1 or ly2, ly3)	273.0 (0.6, >1000)	.076		
Venous invasion (v0, v1 or v2, v3)	4.1 (1.2, 14.0)	.025*	7.4 (1.8, 29.7)	.005*
Intratumoral CD15/CD8 ratio (<2.25 or ≧2.25)	10.8 (3.2, 37.2)	<.001*	14.7 (3.8, 56.8)	<.001*
NLR (<3.07 or ≧3.07)	4.7 (1.4, 15.3)	.011*	5.4 (1.5, 19.6)	.010*
CEA (<5.0 or ≧5.0) (ng/mL)	0.04 (<0.001, 297.2)	.481		
CA19‐9 (<37 or ≧37) (U/mL)	1.3 (0.2, 10.6)	.794		
ALB (<3.8 or ≧3.8) (g/dL)	0.3 (0.1, 1.1)	.066		

Abbreviations: ALB, albumin; CA19‐9, carbohydrate antigen 19‐9; CD, cluster of differentiation; CEA, carcinoembryonic antigen; NLR, neutrophil‐to‐lymphocyte ratio.

* *p* < .05: Intratumoral CD15/CD8 ratio, NLR, venous invasion.

### 3.7 Intratumoral CD15/CD8 ratio predicts the recurrence of gastric cancer in patients with stage II and III tumors

**FIGURE 4:**
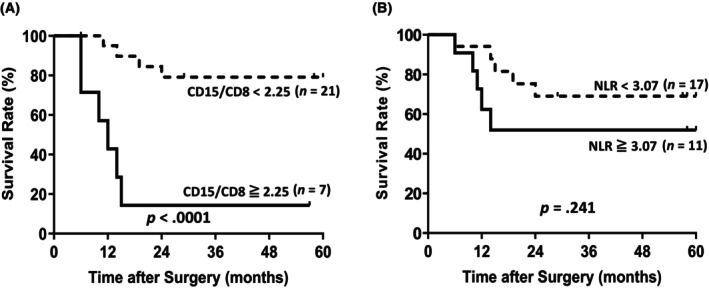
Kaplan–Meier curve showing RFS of patients with stage II‐III gastric cancer. (A) RFS based on the CD15/CD8 ratio (*p* < .0001). (B) RFS based on the NLR (*p* = .241) (*n* = 28). CD, cluster of differentiation; NLR, neutrophil–lymphocyte ratio; RFS, relapse‐free survival.

Only patients with stage II and III cancer who were candidates for postoperative adjuvant chemotherapy were included in the analysis. The RFS of patients with a high intratumoral CD15/CD8 ratio was significantly worse than that of patients with a low CD15/CD8 ratio (Figure 4; *p* < .0001). The five‐year RFS in the low and high CD15/CD8 groups were 81.0% and 14.3%, respectively (*p* < .0001). The HR for RFS was 9.3 (HR: 2.5–34.8, *p* = .001). The five‐year RFS in the low and high NLR groups were 70.6% and 54.5%, respectively (*p* = .241). HR for OS was 2.1 (HR: 0.6–7.2, *p* = .255).

## 4 DISCUSSION

In this study, we investigated the association between recurrence and prognosis in patients undergoing gastrectomy for radical resection of gastric cancer and the ratio of CD15‐positive neutrophils to CD8‐positive lymphocytes (CD15/CD8 ratio) in gastric cancer tissue. This ratio may reflect the immunologic landscape within the gastric cancer tissue. Additionally, we examined the association between the CD15/CD8 ratio and clinicopathological factors and determined the mechanism by which it influences prognosis.

An elevated CD15/CD8 ratio in gastric cancer tissues was associated with poor OS and RFS. Immunohistochemical analyses corroborated the results of comprehensive gene expression analysis using TCGA samples.^3^ This study also supports similar outcomes from previous studies that assessed the proportion of tumor‐infiltrating lymphocytes through immunostaining for CD3, CD8, CD45RO, and CD66b, although those studies did not elucidate the mechanism.^9^

The analysis revealed that gastric cancer tissues with a high CD15/CD8 ratio tended to have more lymphatic invasion, which was significantly associated with more lymph node metastasis. Recent studies have shown that cancer cells alter the immune balance by secreting cytokines and chemokines, inducing endogenous inflammation within the cancer cells, and promoting the metastasis cascade. Animal experiments have demonstrated that such inflammation intensifies with cancer progression, initiating neutrophil‐dependent metastasis.^10^, ^11^, ^12^ Furthermore, the suppression of CD8‐positive cells by infiltrating neutrophils may reduce immune surveillance by lymphocytes, creating an environment that promotes further metastasis. Polymorphonuclear myeloid‐derived suppressor cells (PMN‐MDSCs), known for their potent immunosuppressive capabilities, suppress the number and function of lymphocytes.^13^ PMN‐MDSCs contribute to immunosuppression by depleting arginine, which is essential for lymphocyte proliferation, through the release of arginase 1.^14^ They also produce immunosuppressive mediators, such as reactive oxygen species, peroxynitrites, and prostaglandin E2.^15^ Our findings suggest that a high CD15/CD8 ratio in cancer tissues could signal an immunological environment that promotes lymphatic invasion and lymph node metastasis. Although our study lacks specific immunostaining markers to differentiate PMN‐MDSCs from other neutrophil subtypes, the CD15‐positive neutrophils we observed are considered potential targets for cancer therapy.

This study also assessed the correlation between the CD15/CD8 ratio and NLR (neutrophil lymphocyte ratio) as a prognostic factor in gastric cancer. NLR, reflecting systemic immunologic balance and has long been associated with prognosis.^16^, ^17^ In contrast, the CD15/CD8 ratio was considered more reflective of the local immune status of the tumor and was found to be more predictive of prognosis than NLR. Since these two indices are very weakly correlated in nonparametric analysis did and each functioned as independent prognostic factors, combination them may enhance the accuracy of prognostic prediction. It has been reported that combining several immunological indices can better predict prognosis.^18^ In this study, cases with high levels of either or both NLR and CD15/CD8 ratio experienced recurrence rate approximately 8 times higher than those with low levels of both.

In addition to histopathological factors, HER2 overexpression,^19^ microsatellite instability (MSI),^20^ PDL‐1 expression,^21^ and Ki67^22^ have been reported to predict the prognosis of gastric cancer. MSI and PDL‐1 expression are considered important indicators for predicting the efficacy of immune checkpoint inhibitors.^23^ This evaluation focuses on the aspect of acquired immunity, primarily cytotoxic T cells, but the CD15/CD8 ratio evaluated in this study adds an assessment of the aspect of innate immunity through the measurement of neutrophils. Furthermore, by combining it with the NLR, it is possible to assess both systemic and local immune status, identifying cases with a high risk of recurrence that could not be predicted by existing indicators. Additionally, the method of counting cell numbers to calculate ratios offers the advantages of simplicity and higher quantification compared with assessing staining intensity with immunostaining, making clinical application with a view to automation.

Postoperative adjuvant chemotherapy is the standard of care for stage II and III gastric cancer and is associated with good prognosis.^6^ Stratifying patients at high risk of recurrence and adjusting treatment regimens is critical to further improving outcomes and optimizing patient safety and health care resources. In our study, the CD15/CD8 ratio predicted the risk of tumor recurrence even when analyzed only for stage II and stage III subgroups. Although further validation in more cases is needed, we suggest that the CD15/CD8 ratio, alone or in combination with NLR, has potential as a biomarker for predicting cancer recurrence and for stratifying postoperative adjuvant therapy.

The present study has several limitations. Due to the small number of patients included and the limited number of relapse events observed, further validation in a larger cohort is necessary before the findings can be applied to clinical practice. Additionally, the anti‐CD15 antibody utilized to identify neutrophils in this study also stains other myeloid leukocytes, such as eosinophils, monocytes, and histiocytes. Therefore, it is imperative to employ antibodies capable of specifically identifying these leukocyte subtypes to rigorously assess neutrophil counts. Studies utilizing anti‐CD66b antibodies for the analysis of tumor‐infiltrating neutrophils have been documented, and comparing our findings with these studies is essential. Furthermore, the CD8‐positive cells studied in this study represent a fraction of lymphocytes and do not capture the same aspects as the NLR, which is calculated based on total lymphocyte count. Therefore, it is crucial to explore the optimal combination of leukocyte fractions that accurately represent the immunological landscape of tumor tissue.

## 5 CONCLUSION

In conclusion, our findings suggest that the CD15/CD8 ratio is a predictor of cancer recurrence in patients with gastric cancer, and combining the NLR with the CD15/CD8 ratio could more accurately predict cancer recurrence. A rapid evaluation of the immunological landscape using immunostaining of tumor tissues can provide prognostic information in addition to TNM staging and may assist in establishing an appropriate schedule for detecting recurrence and identifying the need for adjuvant therapy. However, these findings should be validated in larger independent populations.

## AUTHOR CONTRIBUTIONS

**Junichiro Watanabe:** Conceptualization (equal); data curation (lead); formal analysis (lead); investigation (lead); methodology (equal); resources (equal); software (equal); validation (equal); visualization (lead); writing – original draft (lead); writing – review and editing (lead). **Takashi Kimura:** Conceptualization (lead); formal analysis (equal); methodology (lead); supervision (equal); validation (equal); visualization (equal); writing – original draft (equal); writing – review and editing (equal). **Zenichiro Saze:** Conceptualization (lead); formal analysis (equal); methodology (lead); supervision (equal); validation (equal); visualization (equal); writing – original draft (equal). **Naoya Sato:** Conceptualization (equal); formal analysis (equal); validation (equal). **Yasuhide Kofunato:** Formal analysis (equal); validation (equal). **Teruhide Ishigame:** Formal analysis (equal); validation (equal). **Ryo Okada:** Formal analysis (equal); validation (equal). **Akira Kenjo:** Formal analysis (equal); validation (equal). **Koji Kono:** Formal analysis (equal); supervision (equal); validation (equal). **Shigeru Marubashi:** Conceptualization (equal); formal analysis (equal); project administration (lead); supervision (equal); validation (equal).

## CONFLICT OF INTEREST STATEMENT

The authors have stated explicitly that there are no conflicts of interest in connection with this article.

## ETHICS STATEMENT

This study was performed in line with the principles of the Declaration of Helsinki (2013). The experimental protocols were reviewed and approved by the Fukushima Medical University Ethics Committee (approval number: 2022, July 2, 2014). The study was performed in accordance with the ethical guidelines of studies on medical science for humans (Ministry of Education, Culture, Sports, Science and Technology, Ministry of Health, Labor and Welfare Notification No. 3) announced in February 2017.

## Data Availability

The authors confirm that the data supporting the findings of this study are available within the article.
